# Recent active rifting on Venus revealed by wide rift flank uplifts

**DOI:** 10.1038/s41561-026-02044-8

**Published:** 2026-07-24

**Authors:** Xi Yang, Taras V. Gerya, Anna J. P. Gülcher

**Affiliations:** 1 Department of Earth and Planetary Sciences, ETH Zürich, Zurich, Switzerland; 2 Geosciences Department, University of Freiburg, Freiburg, Germany; 3 Center for Space and Habitability, University of Bern, Bern, Switzerland

**Keywords:** Tectonics, Geodynamics

## Abstract

Rifts are prominent tectonic features on Venus, yet their formation, evolution and tectonic activity remain debatable. Here we conducted 3D numerical thermomechanical experiments with visco-elasto-plastic compressible rock rheology to systematically investigate the rifting dynamics and topography under Venusian conditions. To compare active and inactive rift morphologies, the relaxation of rift topography after extension ceases was also investigated. Our modelling results suggest a relatively thick thermal lithosphere and a strong crust with dry diabase or mafic granulite rheology in rifted regions and demonstrate that wide rift flank uplifts strongly indicate either presently ongoing Venusian rifting or activity within the past few tens of millions of years. The rift valleys in the Ganis, Dali and Devana Chasmata have wide rift flank uplifts, and their characteristic topographic features are comparable to the modelled early-stage active rifts undergoing relatively high extension rates of 3−10 cm yr^−1^. This suggests that the Venus rifts in these regions are still active or were active very recently. Their morphology also indicates faster extension rates than commonly assumed for Venusian rifting, potentially associated with recent vigorous mantle plume activity.

## Abstract (web summary)

Comparisons between thermomechanical models and Venusian rift morphologies indicate that rifts on Venus are still, or were recently, active and may have undergone faster extension than usually assumed, potentially because of mantle plume activity.

## Main

Extensional rift systems, which are also termed ‘chasmata’, described by their elongated, deep, steep-sided morphology, are widely distributed on Venus, providing important insights into Venus’s tectonic history and activity. Global observations from the National Aeronautics and Space Administration (NASA) Magellan mission suggest no evidence of plate tectonics on Venus, although it shares major physiological similarities with Earth^1^. Understanding Venusian tectonics offers key insights into the evolution of terrestrial tectonic processes.

Rift zones on Venus are estimated to have a total length of ~40,000 km and cover an area of ~8% of the Venusian surface^2^. Most rift zones are stratigraphically among the youngest formations^3^. Some of the major rifts connect to prominent topographic rises^4,5^, which are suggested surface expressions of deep-seated upwelling mantle plumes^4,6^. Furthermore, many coronae (circular tectono-magmatic features) are clustered around some rifts^7,8^. Despite these observations, the origins and current state of Venus’ rift zones remain debatable^9^.

Outstanding questions for Venusian rifts relate to their physical mechanisms and controlling parameters. Previous studies have documented the similarity of topographic features between Venusian rifts and terrestrial continental rifts (for example, refs. ^10,11^) and oceanic ridges (for example, refs. ^12,13^) yet they have clear differences. Venusian rifts are generally wider than rifts on Earth, which could be explained by the unstable extension of a strong upper crustal layer^14^ and/or the absence of sedimentary processes^11^. In contrast to the thoroughly studied terrestrial rifts (for example, ^15–17^), few studies focused on the dynamics of Venusian rifts.

Until now, physical controls of Venus rifts have been explored using theoretical analyses^18^, simple stretching models^19^, 2D^7,20^ and 3D^21^ thermomechanical models. The 2D models by Regorda et al.^20^, which use limited tectonic extension rates (1cm yr^−1^) and a very thin lithosphere, indicate strong crustal rheology needed for rift localization and further show that the rifting process is critically affected by crustal thickness. Gülcher et al.^21^ showed different types of 3D rifting morphologies with a 2-cm yr^−1^ extension rate that depends on crustal rheology, crustal thickness and lithosphere thermal structure. However, both studies used a simplified visco-plastic incompressible rock rheology and no self-consistent grain-size evolution. Furthermore, none of the previous studies investigated the topography evolution after the extension ceases, which is critical for distinguishing active and fossil rifts because the stresses may be short-lived without plate tectonics on Venus.

Here, we investigate rifting on Venus using 3D thermomechanical models with realistic visco-elasto-plastic rheology and grain-size evolution. We explore a wide range of extension rates beyond the commonly assumed low extensional rates for Venus. Moreover, we model long-duration relaxation after extension ceases. We find that a crust of strong mafic rheology with an initially 150-km-thick lithosphere, which undergoes a rapid but short extension episode, can produce key Venus rift morphologies. Our results suggest that parts of the Ganis, Dali and Devana Chasmata are presently or very recently active.

## Numerical results for tectonically active rifts

**Extended Data Fig. 1:**
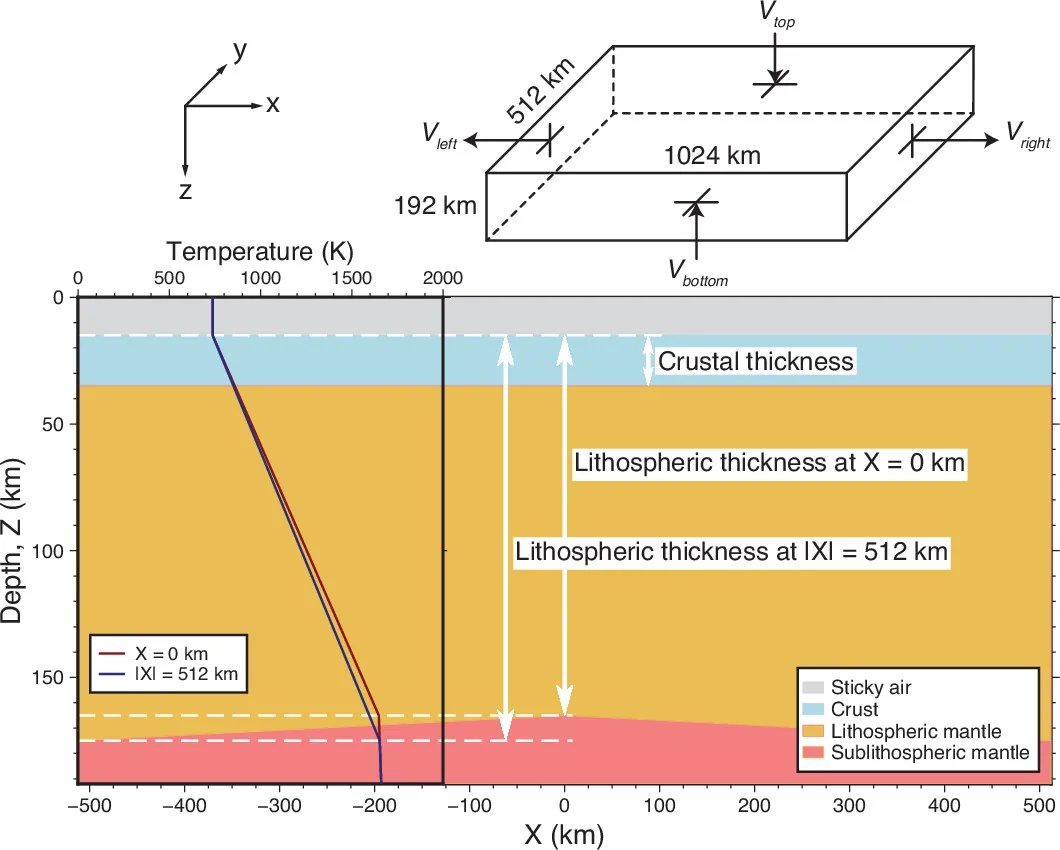
Initial model setup and boundary conditions for numerical experiments. The upper row shows the direction of the three axes, model extension, and the velocity boundary conditions. The front and back boundaries in the x-z plane use the free slip conditions, and the rest have velocity boundary conditions according to the extension rate (see Methods). The lower figure shows the x-z profile of the initial setup with a 15-km-thick sticky air (gray), a 20-km-thick crust (blue), and a lithosphere (orange) with a thickness of 150 km in the middle (X = 0 km). The red and blue curves show the initial temperature profiles at X = 0 km and ∣X∣ = 512 km, respectively. All models are uniformly set up along the y-axis.

Using a state-of-the-art thermomechanical model that accounts for visco-elasto-plastic compressible rock rheology and plastic strain-induced weakening (Methods), we systematically explore the effects of thermal lithosphere thickness, crustal rheology and tectonic extension rate on rifting structures under Venus conditions (Extended Data Table 1). In particular, we test models with three different rheologies for the 20-km-thick crust with an assumed basaltic/gabbroic composition^22^: plagioclase, dry diabase and mafic granulite (Extended Data Table 2). The mantle material uses dry olivine rheology^23^ that accounts for grain size evolution (Methods). We model the rifting with slow, moderate and fast extension rates of 1, 3 and 10 cm yr^−1^, respectively. To initialize spontaneously localizing rifting, we prescribe a 150-km-thick thermal lithosphere (~1,650 K isotherm) that is slightly warmer, and thus thinner, in the middle of the model (Extended Data Fig. 1).

For the topographic features of the rift (Extended Data Table 3), we focus on the width of the rift valley, the elevation offset from peak to bottom of the rift valley and the width of the rift flank uplift, defined as extending from the peak outwards to where the flank uplift is indistinguishable from the surrounding terrain in topography. For all modelled and observed rifts, these features are extracted with a lateral resolution of 16 km, consistent with the global resolution of the Magellan measurements (~10–25 km).

**Fig. 1:**
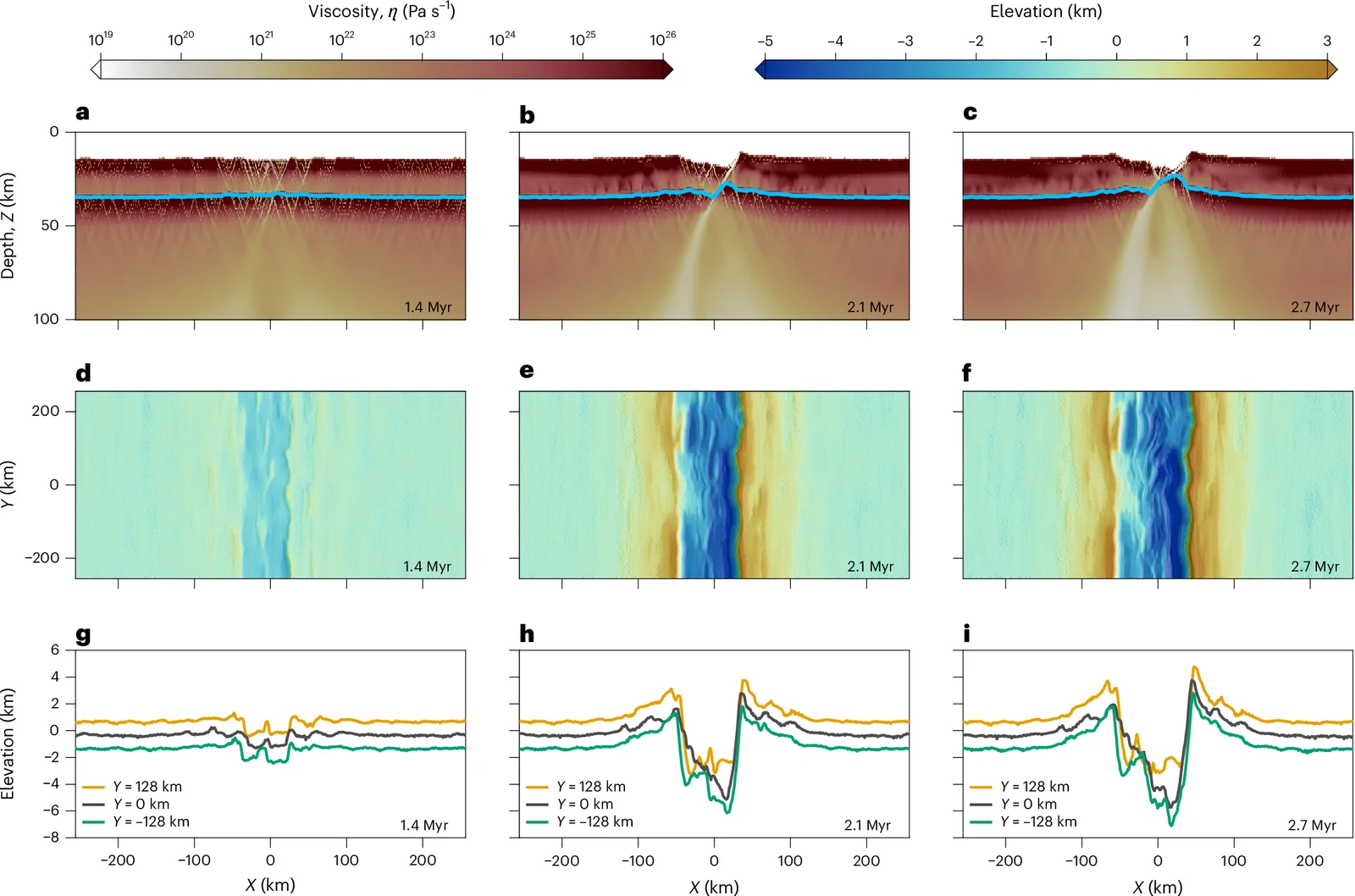
Reference model of rift evolution on Venus. The displayed model has a granulite crust and a moderate extensional rate of 3 cm yr^−1^ (model G2 in Extended Data Table 1). **a**–**i**, From top to bottom, the rows correspond to viscosity profiles at *Y* = 0 km, with the crust–mantle interface (Moho) shown in blue (**a**–**c**), surface topography (**d**–**f**) and topographic profiles at *Y* = 0 (grey), −128 (orange) and 128 (green) km, respectively (**g**–**i**). To improve visibility, the topographic profiles at *Y* = ±128 km have a ±1-km vertical offset, respectively. From left to right, the columns correspond to model ages of 1.4 (**a**,**d**,**g**), 2.1 (**b**,**e**,**h**) and 2.7 (**c**,**f**,**i**) Myr as shown in the bottom right.

We show the reference model G2 with the strongest mafic granulite crust and a moderate extension rate (3 cm yr^−1^) to illustrate the development of Venusian rifting. In the early stage, the strain is localized in multiple conjugate normal faults (Fig. 1a) and forms a shallow graben on the surface (Fig. 1d,g). The topography of mature rifts generally depends on the pattern of early strain localization. In this model, it controls the rapid formation of a dominant steep normal fault with a large elevation offset (Fig. 1b,e,h), which subsequently evolves into a low-angle detachment fault (Fig. 1c,f,i).

**Fig. 2:**
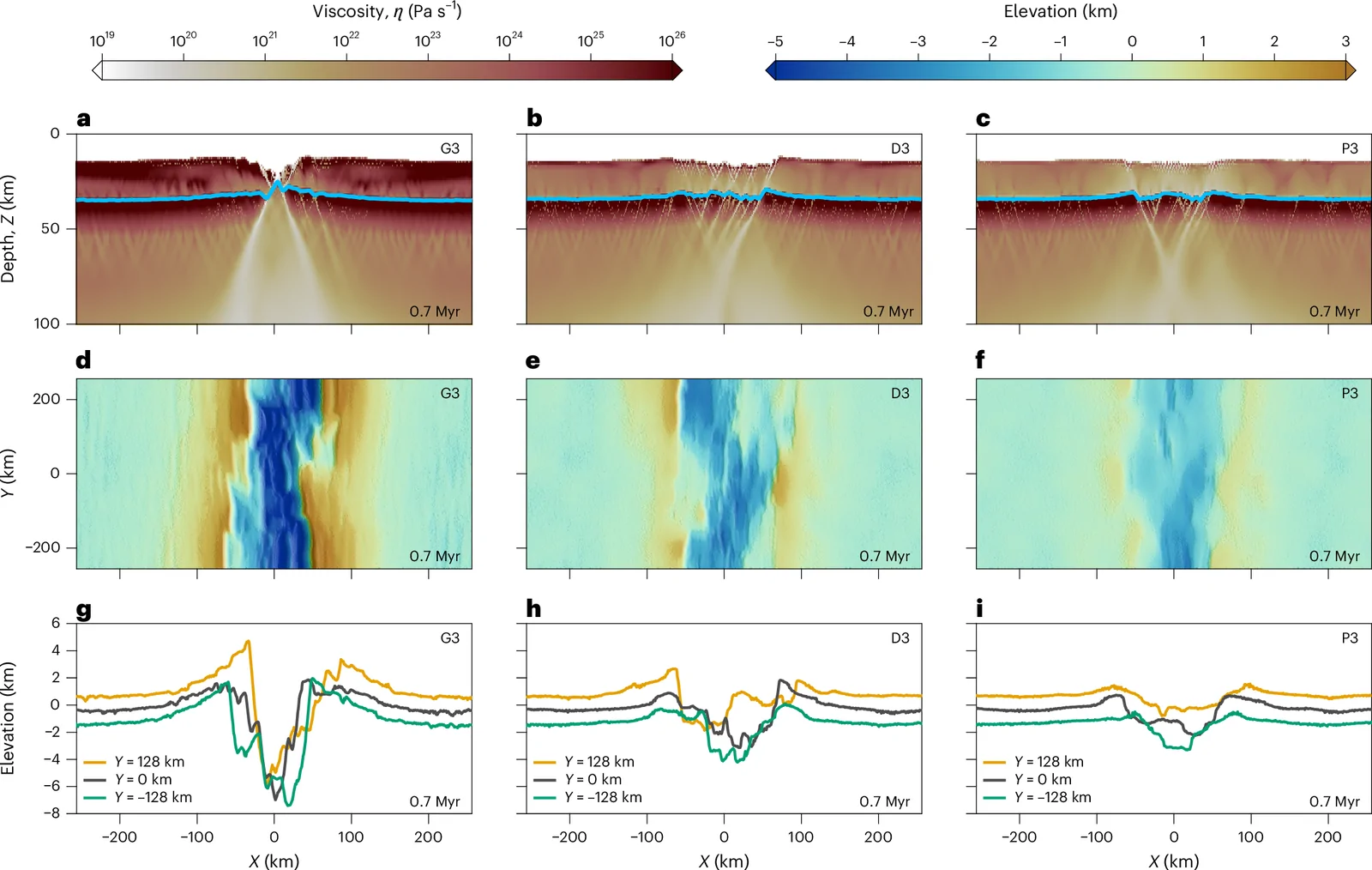
Effect of crustal rheology on modelled rifts. **a**–**i**, From left to right, the columns correspond to models subjected to the same fast extensional rate of 10 cm yr^−1^ with the crustal rheology of mafic granulite (**a**,**d**,**g**), dry diabase (**b**,**e**,**h**) and plagioclase (**c**,**f**,**i**), respectively (models G3, D3 and P3 in Extended Data Table 1, denoted in the top right). The results are displayed and annotated in the same way as in Fig. 1.

The reference model represents a Venus rift formed with granulite crust. To show the effects of crustal rheology, which affects the brittle layer thickness and the plastic deformation in the crust, we compare models subjected to a fast extension rate of 10 cm yr^−1^ (Fig. 2). The strongest granulite crust (G3) has a thick brittle layer and produces a pair of conjugate dominant normal faults (Fig. 2a) with multiple offsets of the rift axis (Fig. 2d,g). The diabase crust (D3) has a thinner brittle layer and produces a rift valley with several conjugate normal faults and a dominant normal fault at the edge of the rift valley (Fig. 2b). Notably, the steepest slope of the dominant faults changes polarity along large offsets of the rift axis (Fig. 2e,h). The weakest plagioclase crust (P3) undergoes a distributed homogeneous ductile rather than a localized plastic extension. Its rift formation is driven by localization in the mantle (Fig. 2c), forming smaller elevation offsets compared with a stronger crust (Fig. 2f,i).

**Extended Data Fig. 2:**
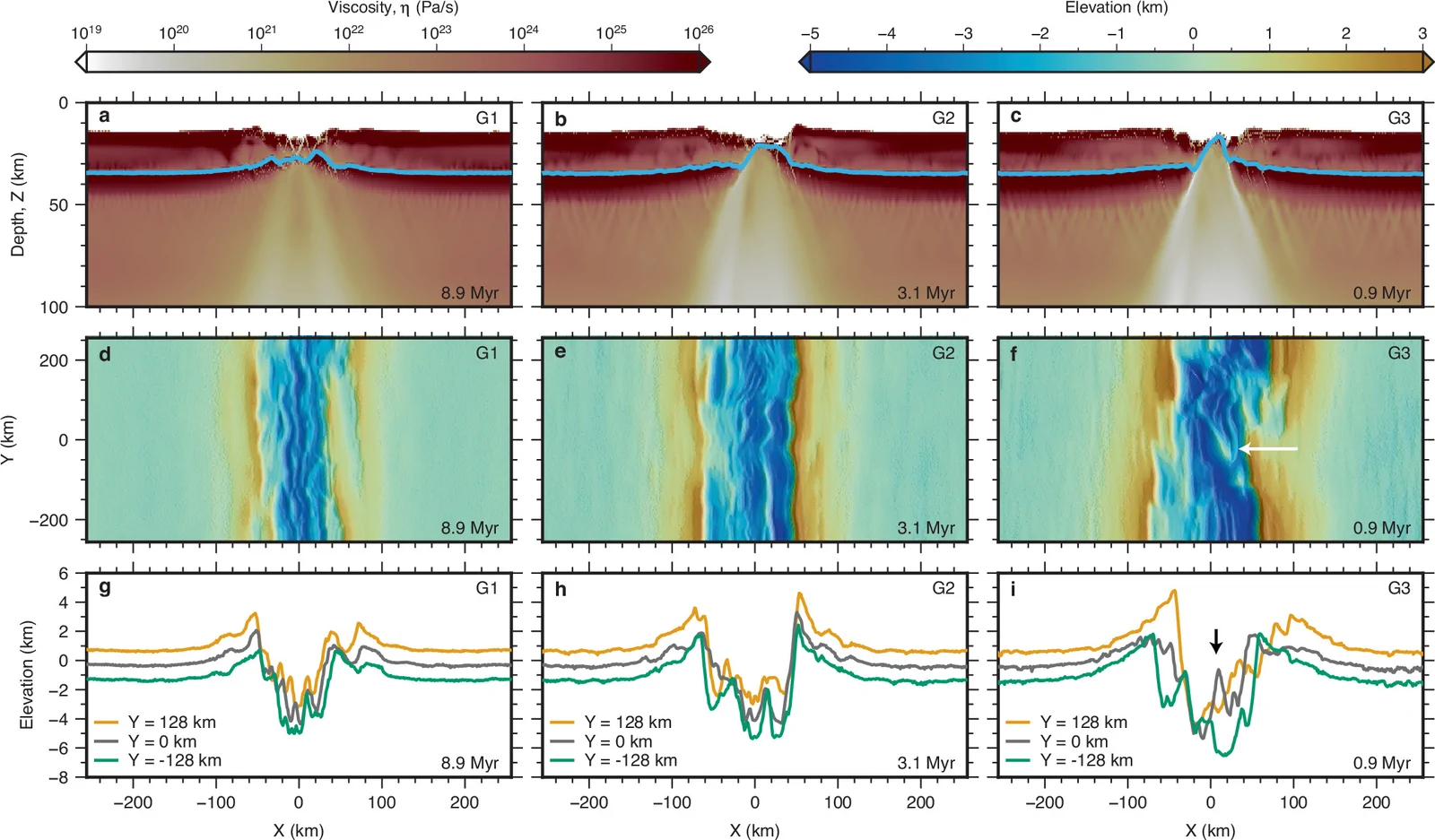
Results of the models with the granulite crust. **a–i**, From left to right, the columns correspond to models G1 (**a,d,g**), G2 (**b,e,h**), and G3 (**c,f,i**), respectively (see Extended Data Table 1). The results are displayed and annotated in the same way as Fig. 1. In panels (**f**) and (**i**), arrows denote the mantle material that is exposed to the surface and forms a topographic high.

The tectonic extension rate particularly influences fault formation during rifting. Granulite crust subjected to moderate (G2; Fig. 1) to fast (G3; Fig. 2) extension rates generate dominant normal or detachment faults (Extended Data Fig. 2). In particular, rifting is more dynamic in model G3, as highlighted by the exposed mantle material on the surface (Extended Data Fig. 2), larger elevation offsets and wider valley and flank uplifts (Extended Data Table 3). In contrast to model G2, the granulite crust subjected to a slow extension rate (G1) only produces several sets of normal faults without a dominant normal fault (Extended Data Fig. 2), as the strain is less efficiently localized with a slower extension.

**Fig. 3:**
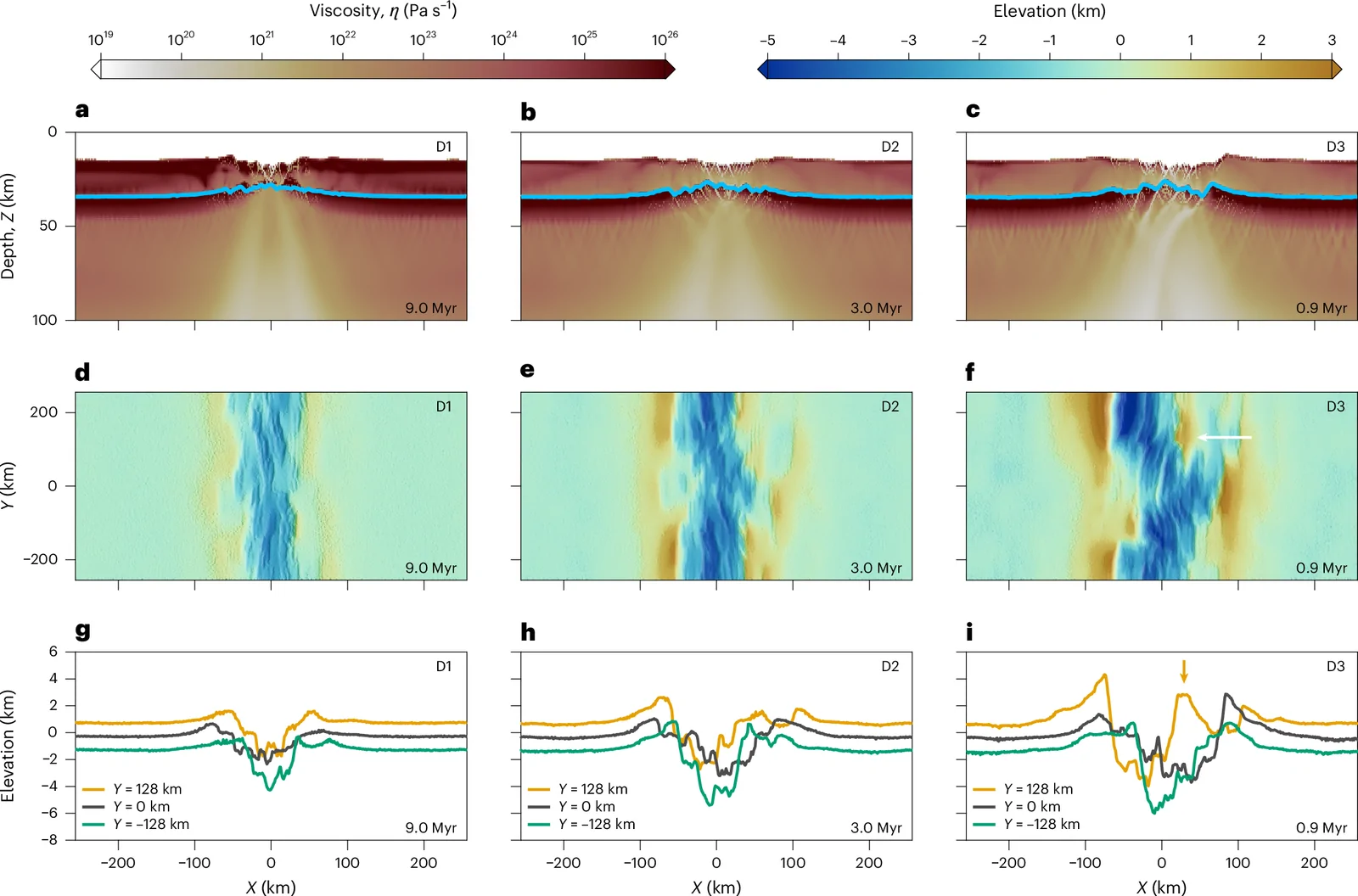
Results of models with a dry diabase crust. **a**–**i**, From left to right, the columns correspond to models D1 (**a**,**d**,**g**), D2 (**b**,**e**,**h**) and D3 (**c**,**f**,**i**), with extension rates of 1, 3 and 10 cm yr^−1^, respectively (Extended Data Table 1). The results are displayed and annotated in the same way as in Fig. 1. In **f** and **i**, arrows denote the newly formed rift flank uplift.

**Extended Data Fig. 3:**
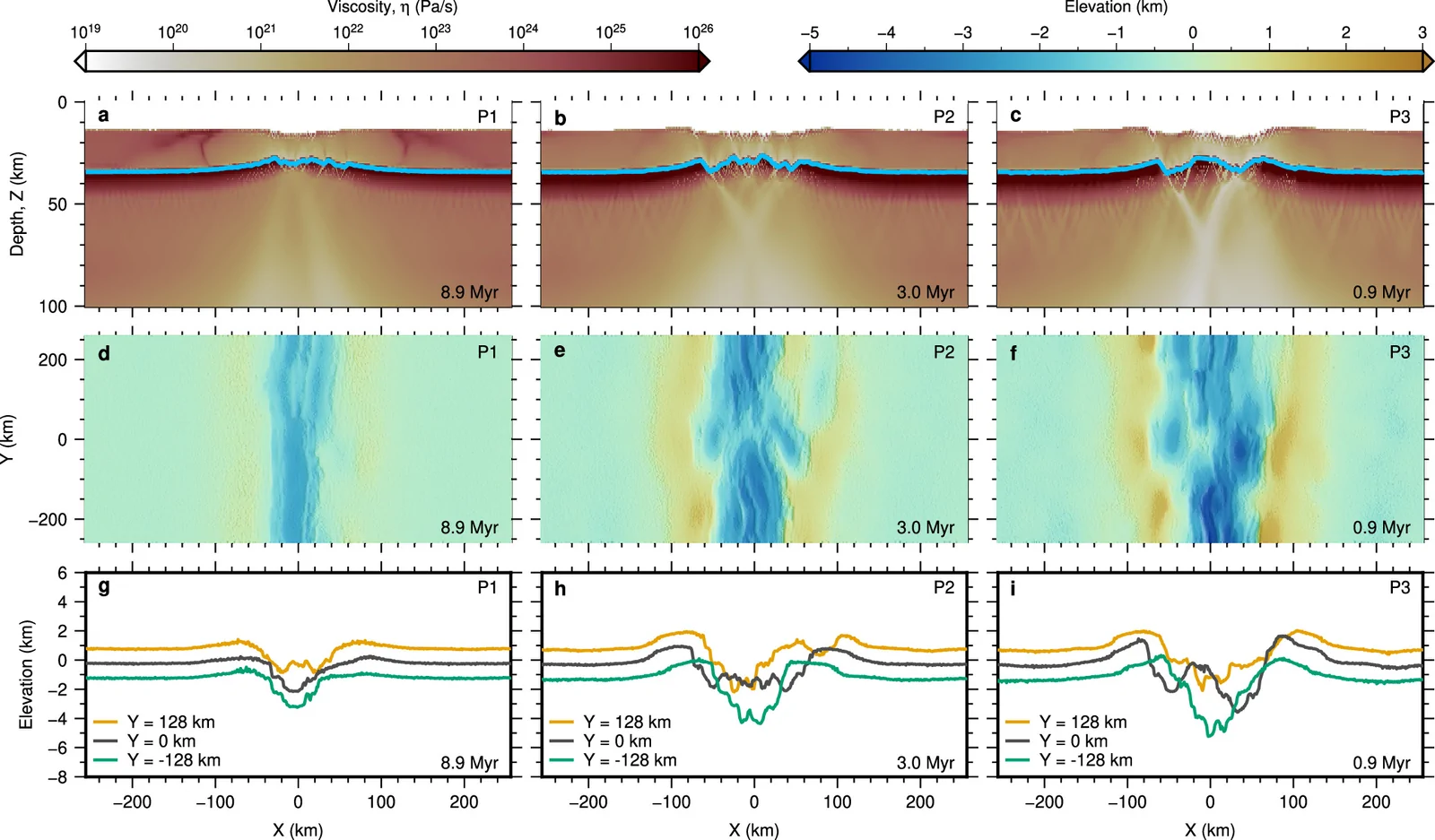
Results of the models with the plagioclase crust. **a–i**, From left to right, columns correspond to models P1 (**a,d,g**), P2 (**b,e,h**) and P3 (**c,f,i**), respectively (see Extended Data Table 1). The results are displayed and annotated in the same way as Fig. 1.

Similarly, the diabase crust subjected to a fast extension rate (D3) develops dynamic and asymmetric rifting (Figs. 2 and 3). A new flank uplift form in the initial valley (Fig. 3f,i), decreasing the median widths of both valley and flank uplifts (Extended Data Table 3). By contrast, the slow extension rate (D1; Fig. 3) produces a rift valley with several small-scale normal faults. The moderate extension rate (D2; Fig. 3) produces a transitional rift that shows elevated flank uplifts with steep valley edges but no dominant normal fault. The weakest plagioclase crust generates a rift valley only when subjected to moderate or fast extension rates (P2 and P3; Extended Data Fig. 3). Fault formation is limited at a slow extension rate (P1) because the predominantly ductile crust cannot efficiently localize strain.

In summary, these models define three rift regimes (Extended Data Table 1) with: (1) no fault-like feature, (2) multiple normal faults but no dominant one or (3) at least a dominant normal fault. Faster extension rates and stronger crustal rheology generally produce larger elevation offsets (median values of about 2–8 km), wider valleys (median width >120 km) and wider flank uplifts (Extended Data Table 3).

**Extended Data Fig. 4:**
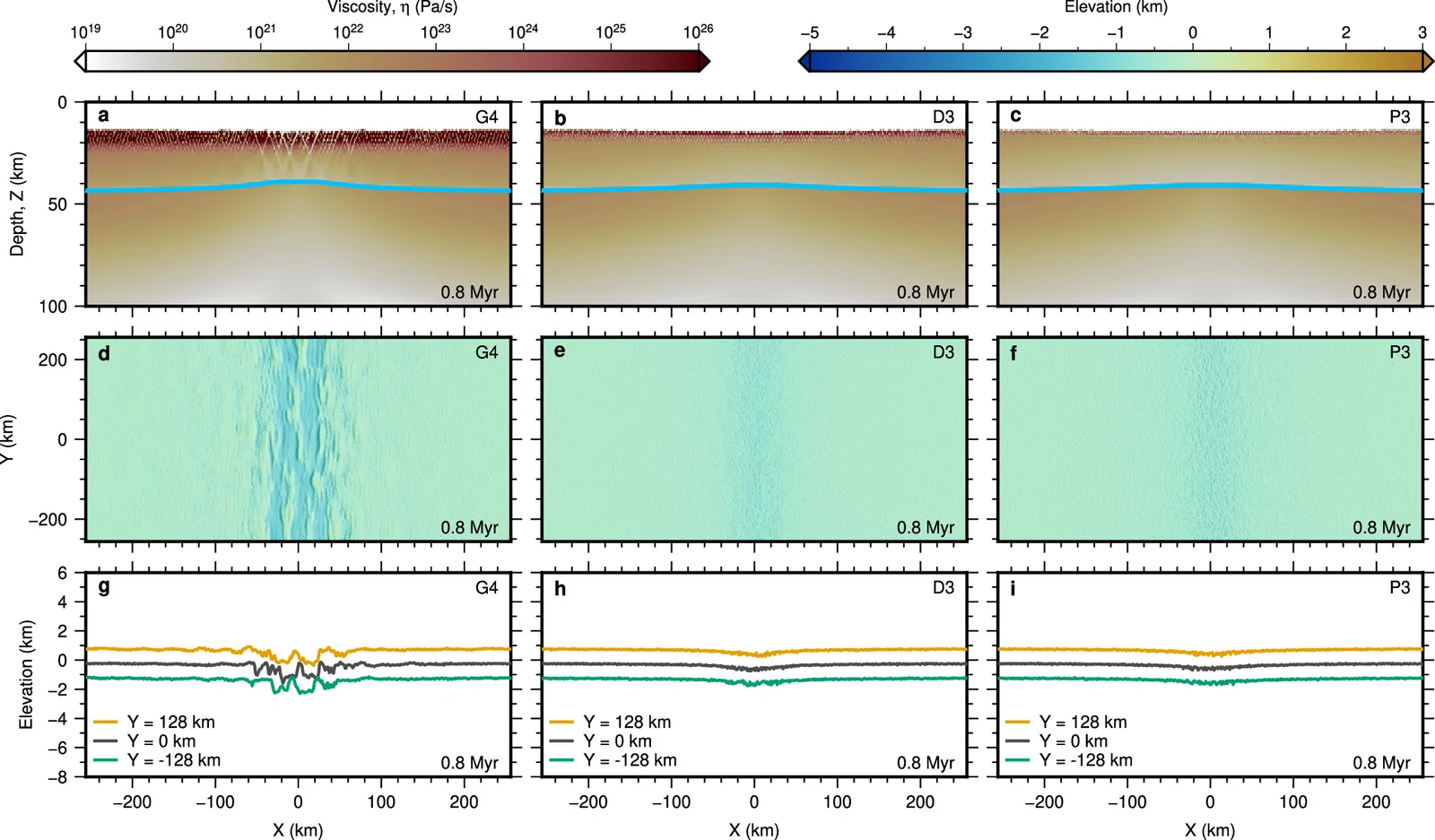
Results of the models with a 100-km-thick lithosphere. **a–i**, From left to right, the columns correspond to models G4 (**a,d,g**), D4 (**b,e,h**), and P4 (**c,f,i**), respectively (see Extended Data Table 1). The results are displayed and annotated in the same way as Fig. 1.

We repeated all models with the highest extension rate of 10 cm yr^−1^ for a 100-km-thick lithosphere (~1,625 K isotherm) with a 30-km-thick crust. Interestingly, none of these models generate a faulted rift valley (Extended Data Fig. 4) because strain localization is limited in the mantle owing to efficient viscosity recovery associated with grain size growth supported by higher temperatures, counteracting grain size reduction and related shear localization. Model G4 with granulite crust forms faults in the crust and wrinkle-like topography, while diabase (D4) and plagioclase crust (P4) cannot produce any rift-like feature. Our results demonstrate that the thermal lithosphere on Venus needs to be perhaps 150-km thick to allow rifting. Given the discrepancies in the definitions of thermal lithosphere (Methods), this finding is comparable to estimates from flexure and geodynamic studies^21,24,25^.

All models with a 150-km-thick initial lithosphere generate wide rift flank uplifts (median width >60 km; Extended Data Table 3). In particular, the strong granulite crust with moderate-to-fast extension rates (G2 and G3) and the diabase crust rheology with a fast extension rate (D3) show median flank uplift widths >120 km. The wide flank uplifts could be indicative of ongoing rifting if their widths decrease rapidly after extension ceases.

## Rift topography relaxation

**Fig. 4:**
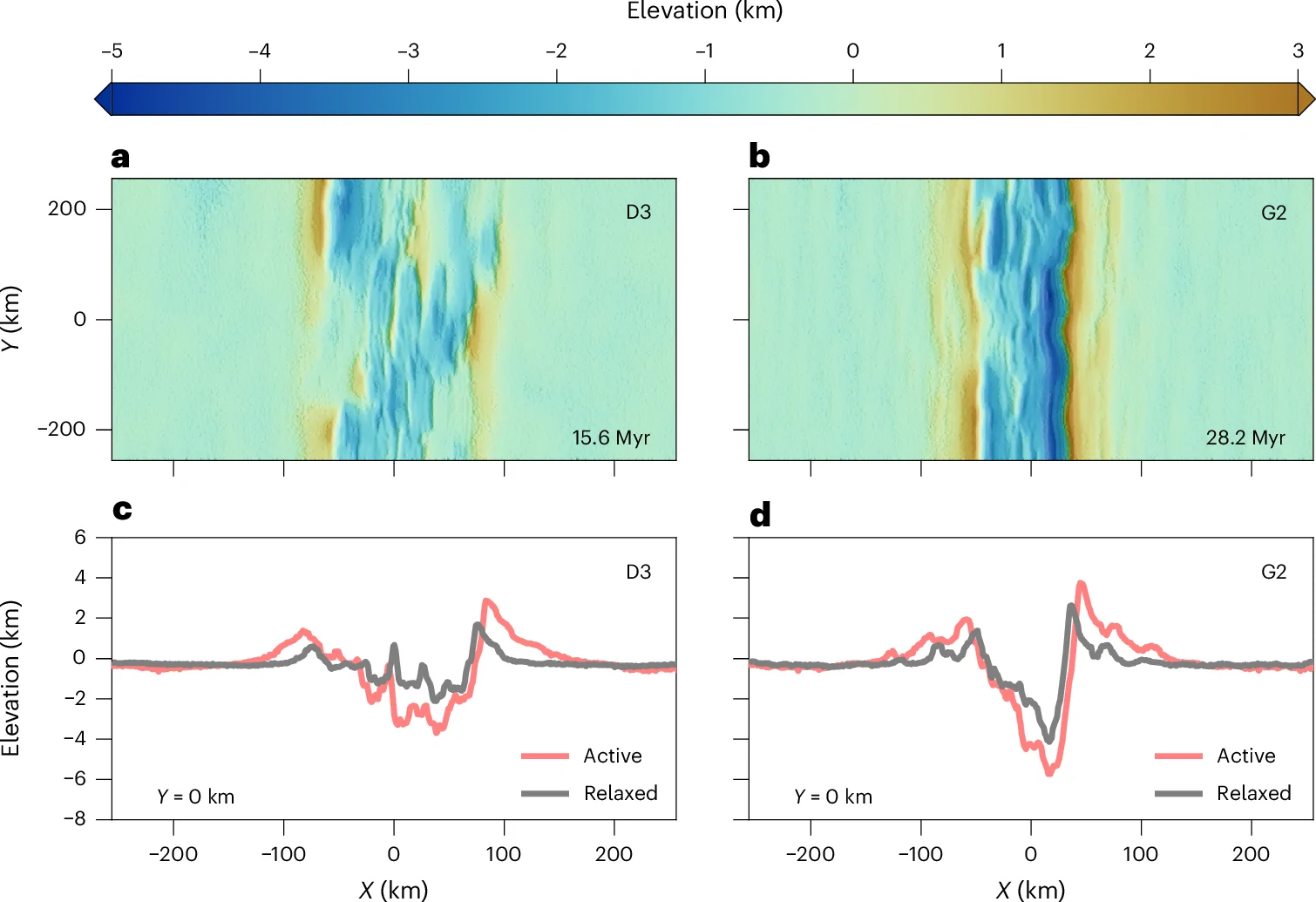
Topography of the relaxed rifts. **a**–**d**, Relaxed surface topography, with model ages shown in the bottom right (**a** and **b**), and the corresponding topographic profiles of active and relaxed rifts at *Y* = 0 km (**c** and **d**) are derived from model D3 with diabase crust subjected to an extension rate of 10 cm yr^−1^ (**a** and **c**) and the reference model G2 with granulite crust subjected to an extension rate of 3 cm yr^−1^ (**b** and **d**). The relaxation starts at 0.7 Myr for model D3 (Fig. 2e) and 2.1 Myr for model G2 (Fig. 1e), and therefore the relaxation times of the shown results are about 15 and 26 Myr, respectively.

To decide whether the wide rift flank uplifts can serve as evidence for active rifting, we investigate the isostatic relaxation of rifts by continuing the same numerical models with zero extension rate (Methods). Figure 4 shows the relaxed topography of the rifts in a diabase crust with an initial extension rate of 10 cm yr^−1^ (D3) and a mafic granulite crust with an initial extension rate of 3 cm yr^−1^ (G2), which are the preferred models to compare with observations in the following section. To represent the relaxation of different timescales, we compare the results on a few, a few tens and about 100 Myr.

**Extended Data Fig. 5:**
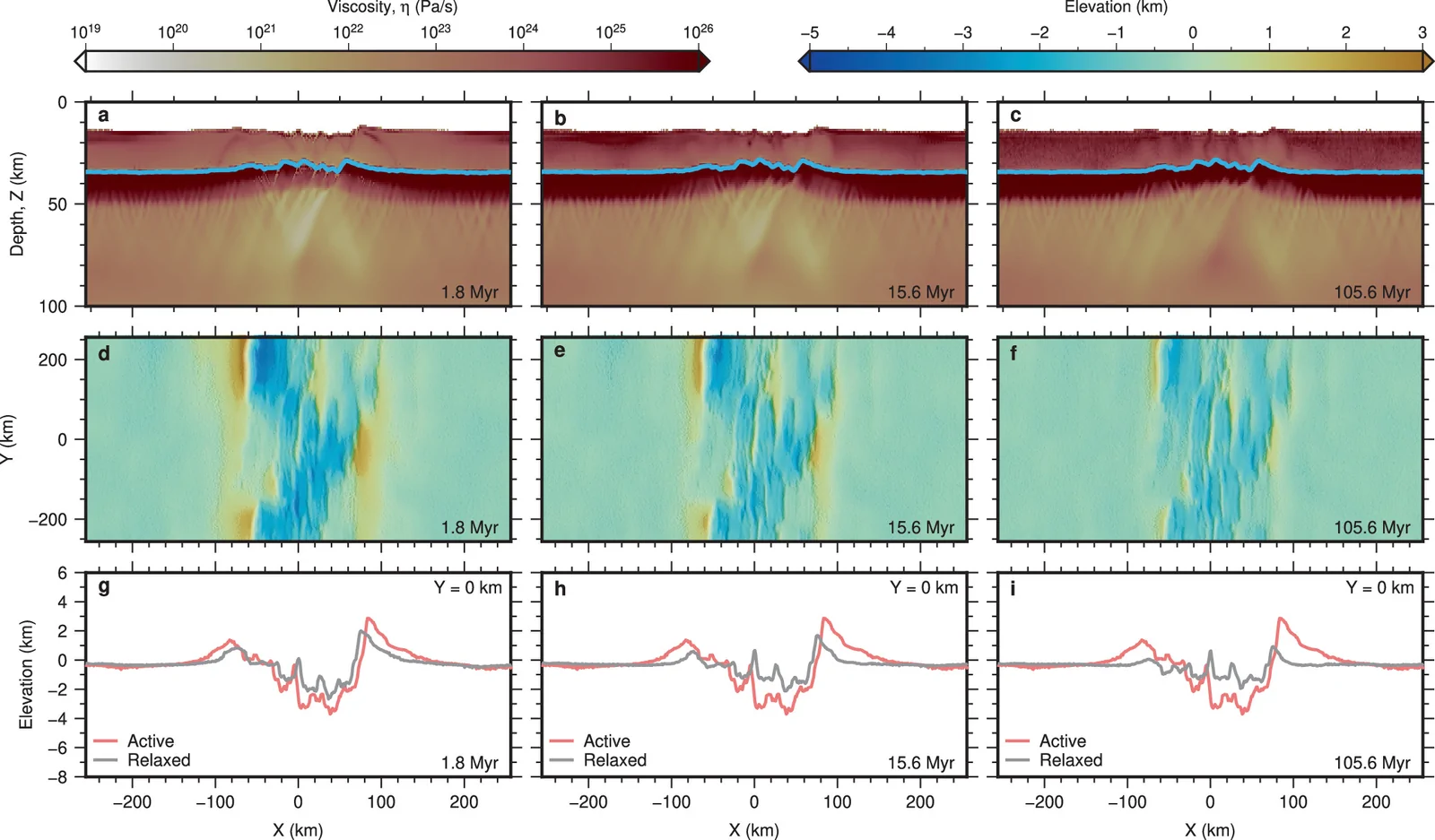
Relaxation of the rift with dry diabase crust (model D3). From top to bottom, the rows correspond to (**a–c**) viscosity profiles at Y = 0 km, with the crust-mantle interface (Moho) shown in blue, (**d–f**) surface topography, and (**g–i**) topographic profiles at Y = 0 km, respectively. The topographic profiles of the active rifts before relaxation and the relaxed rifts are shown in red and gray, respectively. The model ages are shown at the bottom right. The relaxation starts at 0.7 Myr model age (see middle column of Fig. 2). The relaxation times are about (**a,d,g**) 1, (**b,e,h**) 15, (**c,f,i**) 105 Myr from the left to right columns, respectively.

The rift developed in the dry diabase crust is strongly affected by isostatic relaxation (Fig. 4a,c and Extended Data Fig. 5). The median widths of the flank uplifts decrease from 120 km to <90 and <40 km after about 1 and 15 Myr of relaxation, respectively (Extended Data Table 3). After ~105 Myr of relaxation, the flank uplifts have mostly disappeared, with a median width of <30 km, except for the surface expression of the dominant normal faults at the edge of the rift valley (Extended Data Fig. 5f,i). Its median elevation offset decreases by ~60%, and the rift valley becomes slightly narrower.

**Extended Data Fig. 6:**
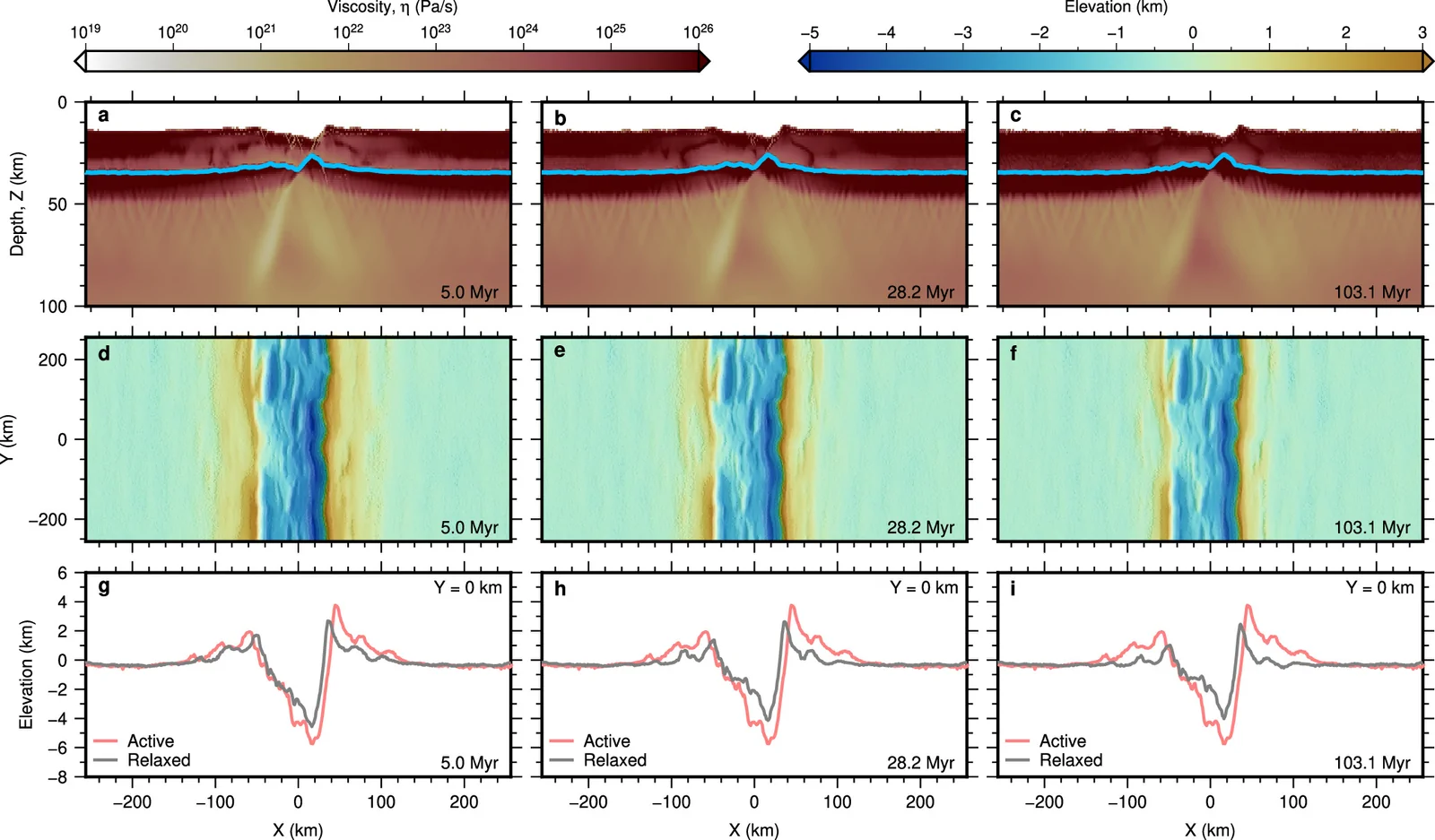
Relaxation of the rift with granulite crust (model G2). The relaxation starts at 2.1 Myr model age (see middle column of Fig. 1). The relaxation times are about (**a,d,g**) 3, (**b,e,h**) 26, (**c,f,i**) 101 Myr from the left to right columns, respectively. The results are displayed and annotated in the same way as Extended Data Fig. 5.

Most of the topographic features of the rift in the mafic granulite crust (G2) remain preserved after relaxation of ~26 Myr (Fig. 4b,d and Extended Data Fig. 6). The width of the flank uplift decreases from ~130 km to 80 and 60 km after about 3 and 26 Myr of relaxation, respectively (Extended Data Table 3). After ~100 Myr of relaxation, the median width of the flank uplifts decreases further to about 50 km (Extended Data Fig. 6f,i). However, the dominant detachment fault can still be identified in the topography, with an median elevation offset decreasing by ~30%. This is not surprising, as mafic granulite is the strongest tested crustal rheology with the highest activation energy (Methods), which controls the longevity of isostatic relaxation by ductile crustal deformation under topographic stresses.

The rift topography after relaxation of both investigated models shows distinctly different topographic morphologies from active rifting stages. These combined results demonstrate that the wide rift flank uplifts (≳100 km; Extended Data Table 3) are indeed a signature of currently or recently active rifting.

## Observational evidence of active rifts on Venus

**Fig. 5:**
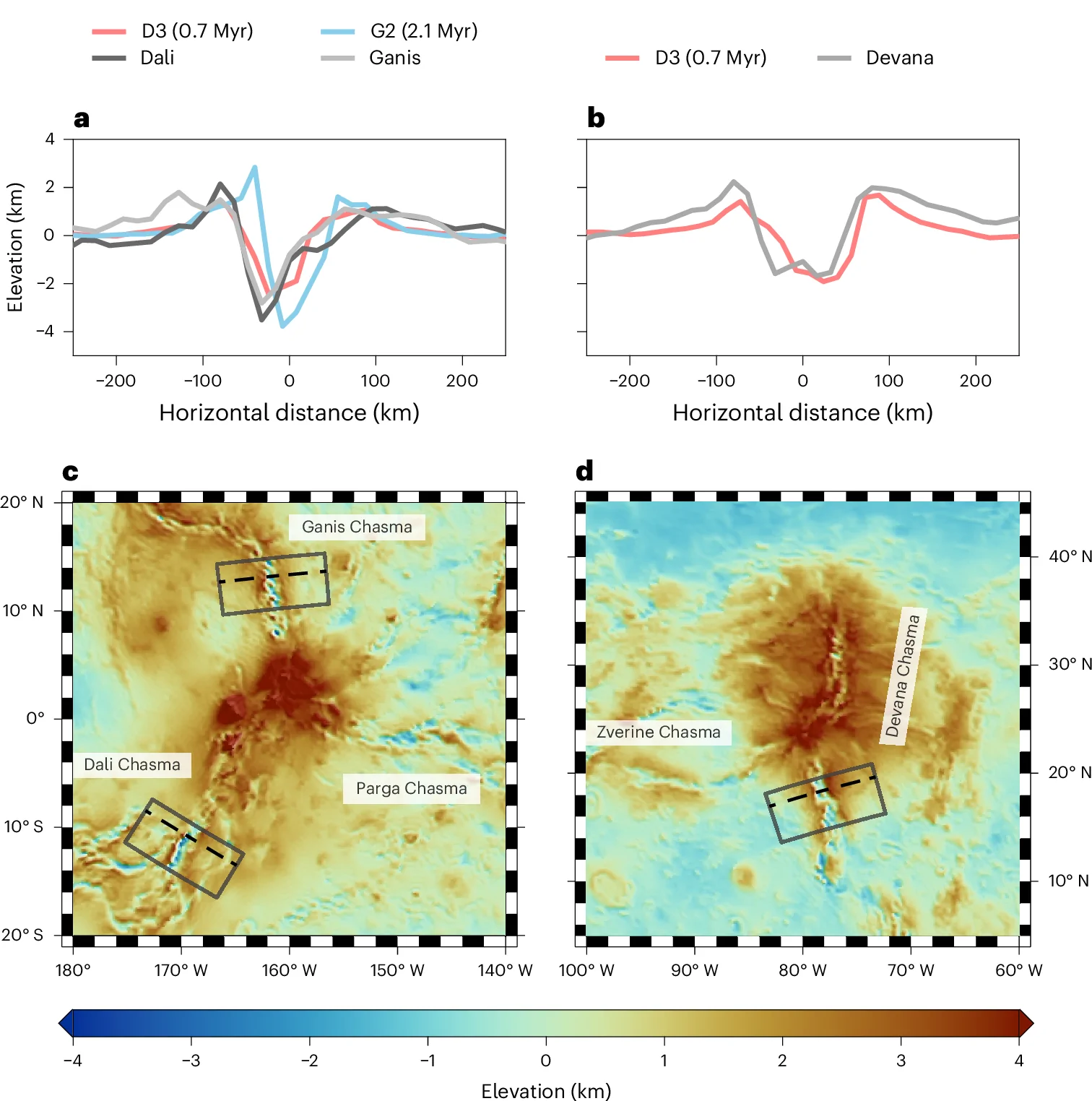
Comparison of the modelled active rifts with observations of Venusian rifts in the Atla and Beta Regiones. **a**–**d**, Topographic profiles extracted from models D3 (*Y* = −120 km (**a**) and 8 km (**b**)) and G2 (*Y* = 72 km) are downsampled to a 16-km resolution (Methods) and compared with observations. Regional maps of Atla (**c**) and Beta (**d**) Regiones with the corresponding observed profiles in **a** and **b**, respectively, are extracted from the VenusTopo719 model^26,45^. Dashed lines indicate the profile locations, oriented from west to east. The boxes denote the regions used to extract the topographic features (Extended Data Fig. 7a–c). The regional maps are shown using a cylindrical equidistant projection.

To compare the models with observations, we focus on the straight segments of the rift valleys that are characterized by clearly expressed deep individual troughs^5^. The topography of three rift valleys from Ganis, Dali and Devana Chasmata (Fig. 5) is extracted from the Venus topography model^26^. For each valley, profiles are extracted with the same lateral extent as the models (Fig. 5c,d).

**Extended Data Fig. 7:**
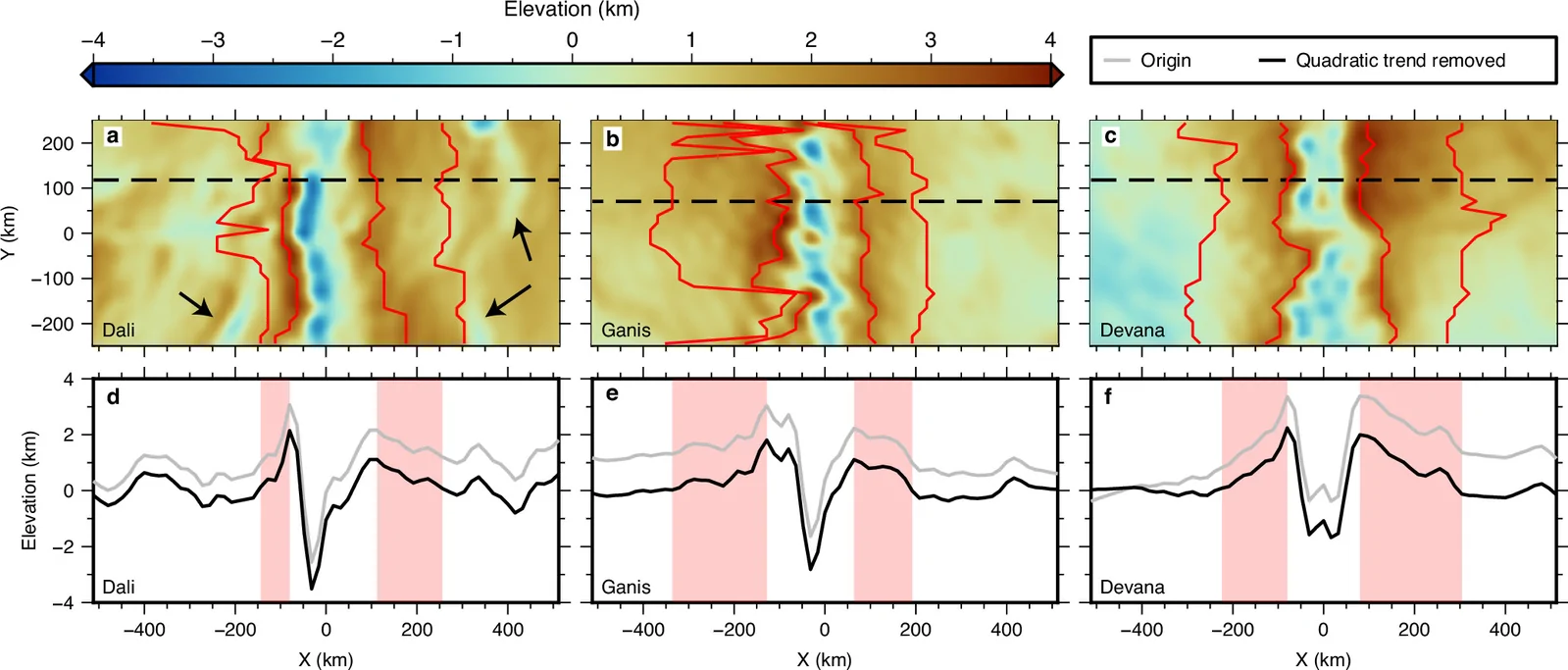
Topography of the Venusian rift valleys. (**a–c**) Topographic map and (**d–f**) profiles of the Dali, Ganis, and Devana Chasmata are derived with a sampling size of 16 km, which are extracted from the VenusTopo719 model (ref. ^26^). The coordinates of the shown maps start from west (x axis) and south (y axis), and their corresponding regions are shown in Fig. 5. The profile locations are indicated by dashed lines. The origin and the detrended profiles (the same as in Fig. 5) are shown in gray and black, respectively. The rift flank uplifts (see Method) are highlighted by red. In panel (**a**), arrows denote the nearby grabens with smaller topographic offsets.

In the Atla Regio (Fig. 5c), Dali Chasma (12° S, 170° W) shows a steep slope with a large elevation offset of ~5 km on the west side of the valley, while the east side shows a gentle slope (Fig. 5a), consistent with the single dominant normal or detachment fault. The flank uplifts of Dali Chasma are narrower on the west than on the east (Extended Data Fig. 7a), with a median average width of about 110 km (Extended Data Table 3), perhaps affected by the nearby grabens with smaller elevation offsets^5^ that are not comparable to our models of active rifting.

Ganis Chasma (13° N, 162° W) shows wide flank uplifts with a median width of about 160 km (Extended Data Fig. 7b and Extended Data Table 3). In contrast to the Dali Chasma, the Ganis Chasma presents stronger horizontal offsets in the rift flank uplifts, with widths varying by more than 100 km. The west side of the rift valley shows a steep slope with an elevation offset of ~4 km, while the opposite east side is relatively gentle (Fig. 5a), consistent with the presence of a dominant normal fault.

In the Beta Regio (Fig. 5d), Devana Chasma (17° N, 78° W) shows wide flank uplifts with a median width exceeding 180 km (Extended Data Fig. 7c and Extended Data Table 3). In contrast to Dali and Ganis Chasmata, the rift valley in Devana Chasma shows steep slopes on both sides and a flat valley bottom (Fig. 5b), making it incomparable to our models. Nevertheless, the presence of a wide rift flank uplift suggests that Devana Chasma is a recently or currently active rift.

The topographic features of these rift valleys, in particular their wide flank uplifts, are comparable to our models of active rifts in a strong dry diabase or mafic granulite crust with a relatively high extension rate of 3–10 cm yr^−1^ after 60–70 km of total extension (Fig. 5a,b). Plagioclase crust models generate rift flank uplifts with a median width <100 km (Extended Data Table 3) without a steep offset corresponding to a dominant normal fault, suggesting less consistency with observations than a stronger crust.

## Implications for rifting tectonics on Venus

Our results suggest that the rift valleys of the Ganis, Dali and Devana Chasmata are active presently or recently within a few tens of Myr. This agrees with suggestions of rift activity on the basis of alignment between extensional deformation and the current strain field^27^, global and regional geological mapping efforts^3,28^, flexural modelling studies with high inferred lithospheric heat flux^24^ and active rift-associated volcanism from optical^29^ and radar^30^ observations.

Our models favour dry diabase or mafic granulite crust to generate rifts on Venus, aligned with estimates of basaltic with mafic rheology^11,31,32^ and previous rifting models^20,21^. Our models further predict that a dry diabase crust generates rifts that are best comparable to Venus’ observations when the remarkably high extensional rate is 10 cm yr^−1^, comparable to fast terrestrial continental rifting resulting from lithosphere weakening^33^. The suggested regions of weak lithosphere on Venus^34,35^ may help accelerate rifting. By contrast, if the extension rate is moderate at 3 cm yr^−1^, our results prefer the mafic granulite crust. This agrees with the constraint that the continental crust on Venus should have granulite rheology^36^.

Future 3D numerical investigations of Venus rifts will be needed to address deficiencies of our simplified homogeneous lithosphere model with unidirectional extension. First, our models do not consider any expected heterogeneity of the lithosphere. For example, Phoebe Regio likely exhibits variations in crustal thickness exceeding 10 km (ref. ^37^). The heterogeneity of the Venus lithosphere (for example, refs. ^25,38^), and stronger strain weakening of rocks, can result in faster strain localization and relax the requirement of lithosphere thickness. However, the thickness of the thermal lithosphere is unlikely to be less than 100 km, as the strain cannot efficiently localize in the mantle in this case.

Second, our models did not consider complex driving mechanisms of rifting, particularly mantle plumes and multidirectional extension. The preferred relatively high (3–10 cm yr^−1^) extension rates for Venus rift formation may be explained by recent vigorous mantle plume activity^39,40^, in line with volcanic rises and buoyant mantle material revealed by thermal^6^ and gravity^41^ signals associated with rifts. Moreover, the chasmata show dominant triple junctions in the Atla and Beta Regiones (Fig. 5c,d). Previous terrestrial 3D geodynamic models showed that triple and quadruple junctions can be formed by multidirectional extension^42^, and plume-induced rifting combined with multidirectional extension has been proposed for the Central African rift system^43^. It is reasonable to assume that the rifting on Venus operates under comparable mechanisms (for example, ref. ^13^). The interaction between a mantle plume and rift with unidirectional extension was explored in Gülcher et al.^21^ but could not form common morphologies of coronae embedded within Venus rifts. This suggests the necessity of exploring multidirectional extension or other far-field extension mechanisms characteristic for Venus-like squishy-lid tectonics^34^ in future studies.

Finally, our models do not include any effect of surface mass wasting that affects topography evolution and the generation of decompression melting (for example, ref. ^17^). Landslides are found to be associated with rift zones^44^, which might explain the wider observed rift valleys and smaller elevation offsets than our models (Extended Data Table 3). However, it remains poorly constrained owing to limited observations^44^.

These important considerations open a broad avenue for future numerical modelling research of Venus rifting, which is crucial for inspiring and assisting targeted observations of the forthcoming Venus missions.

## Methods

### Modelling approach

The 3D thermomechanical numerical code I3ELVIS^46–48^ is used to model the rifting process. It is based on the finite-difference method and the marker-in-cell technique for material advection. The energy, momentum and mass conservation equations are solved in an Eulerian frame, and the physical properties are transported by Lagrangian markers. Non-Newtonian visco-elasto-plastic rheologies and variable temperature-dependent thermal conductivity are applied in the model (Extended Data Table 2).

### Numerical model design

The initial model setup (Extended Data Fig. 1) corresponds to the one used for spontaneous rift initiation (for example, ref. ^49^). The Eulerian computational domain is set as 1,024 × 512 × 192 km^3^ and is resolved with a regular rectangular grid of 1,025 × 129 × 193 nodes on the *X*, *Y* and *Z* axes, respectively. Each Eulerian element is resolved by eight randomly distributed Lagrangian markers. The model is uniform along the *Y* axis, and the profile of the model setups along the *XZ* plane is shown in Extended Data Fig. 1.

The internal free-surface boundary condition on the top of the crust is implemented using a low-density (1 kg m^−^^3^) and viscosity (10^17^Pa s) sticky air with a thickness of 15 km. The large viscosity contrast between air and crust allows topography evolution at the surface with a resolution of a few tens of metres^47^. The crust has a uniform thickness in the model space. The Venusian gravitational acceleration of 8.87 m s^−^^2^ has been used in the model.

Similarly to Gerya^49^, an extensional velocity is applied along the *X* direction to the left and right boundaries and an inwards velocity to the upper and lower boundaries. A free slip condition is applied to the front and back boundaries. The extensional velocity conditions applied at the left and right boundaries are simply half of the extensional rate (*V*_e_). To ensure volume conservation in the model space, compensatory inwards flux is required on the lower and upper boundaries. Their imposed velocities are proportional to the thickness of the rock and the sticky air, thus stabilizing the average vertical position of the surface. This leads to the velocities of $\frac{15}{1024}{V}_{e}$ and $\frac{177}{1024}{V}_{e}$ on the upper and lower boundaries, respectively.

The initial thermal structure and thickness of the lithosphere are defined by prescribing a geotherm uniform along the *Y* direction. An adiabatic temperature gradient of 0.5 K km^−1^ is initially prescribed in the asthenospheric mantle and reaches 1,661.5 K at the lower boundary (*Z* = 192 km). The temperature at the upper and lower boundary is constant, while the rest are the insulated thermal boundaries. Note that the lithospheric thickness is only used to prescribe the temperature as a function of depth and is generally referred to as its value at *X* = 0 km in this paper when describing the model setups. To initialize rifting, we prescribed a lithosphere with a thickness that linearly increases from the middle of the model (*X* = 0 km) to the left and right boundaries (∣*X*∣ = 512 km) by 10 km. The temperature of the sticky air is kept constant at 737 K. For the 150-km-thick lithosphere, the initial temperature at the base is 1,648 K in the middle of the model space and 1,653 K at the left and right boundaries, as defined by the temperature at the lower boundary and the adiabatic temperature gradient. For the 100-km-thick lithosphere, these temperatures are 1,623 and 1,628 K, respectively.

Importantly, we note that the temperature assumed at the base of thermal or ‘mechanical’ lithosphere in our models (~1,650 K) is higher than in other studies, for example, 1,573 K (refs. ^20,21^), 1,300 K (ref. ^39^) or even 1,013 K (ref. ^24^). Our discussed lithospheric thickness values are therefore only apparently larger than the values found in those studies for a similar thermal structure. When considering these discrepancies, our model setup agrees with the estimates for the thermal lithospheric thickness on Venus rifts. For example, 1,013 K corresponds to about 45-km and 31-km depths for the 150- and 100-km-thick lithosphere in our models, respectively, which are consistent with the corresponding global estimates of Venus’ thermal lithosphere^24^ and intrusion-dominated geodynamic model predictions^25^. Given that the thermal lithospheric thickness in our models is comparable to previous studies, we also expect the corresponding elastic thicknesses to be of similar order, that is, <15 km near the rifts^24,25^. Our models yield surface heat fluxes of ~10 mW m^−^^2^ for the initial or inactive rift and tens of mW m^−^^2^ during the active rifting, which are of the same order as those from previous models^20,21^ and observational estimates^24^.

### Governing equations

In vectorial form, the Lagrangian formulation of the governing equations for slowly deforming fully compressible and thermally expanding/contracting heat-conducting media takes the form

1 $$\nabla \cdot {\bf{v}}=-\frac{{\mathrm{D}}\ln \rho}{{\mathrm{D}}{t}}$$

2 $$\nabla \cdot {\boldsymbol{\tau }}-\nabla P=-\rho {\bf{g}}$$

3 $$\rho {c}_{P}\frac{{\mathrm{D}}{T}}{{\mathrm{D}}{t}}=-\nabla \cdot {\bf{q}}+H$$

where *ρ* is density; *c*_*P*_ is the isobaric specific heat capacity; *T* is temperature; *P* is pressure; *H* is the heat source term that includes radiogenic, adiabatic and frictional heating effects; *t* is time; **v**, **g** and **q** are the velocity, gravitational acceleration and heat flux vectors, respectively; and **τ** is the deviatoric stress tensor.

The software accounts for visco-elasto-plastic deformation due to tectonic and gravitational loading and thermal stresses as described in Gerya^47^. The plastic yielding model is regularized by including the viscous damping term^50^. All conservation equations and the plastic yielding condition are solved together in a fully coupled manner by using global Picard iteration combined with a multigrid approach^47^.

### Density model

We use the compressible continuity equation and variable density in the momentum and energy conservation equations. The density of rocks changes with pressure (*P*) and temperature (*T*) according to the equation,

4 $${\rho }_{P,T}={\rho }_{0}\left[1-\alpha (T-{T}_{0})\right]\left[1+\beta (P-{P}_{0})\right],$$

where *ρ*_0_ is the standard density at *P*_0_ = 1 MPa and *T*_0_ = 298 K, and *α* = 3 × 10^−5^ K^−1^ and *β* = 1 × 10^−11^ Pa^−1^ are the coefficients of thermal expansion and compressibility, respectively.

### Visco-elasto-plastic rheological model

The viscous and brittle (plastic) properties (Extended Data Table 2) are implemented via evaluation of the effective viscosity of the material. For the ductile rheology, the contributions from dislocation and diffusion creep are considered by composite rheology for *η*_ductile_

5 $$\frac{1}{{\eta }_{{\rm{ductile}}}}=\frac{1}{{\eta }_{{\rm{diff}}}}+\frac{1}{{\eta }_{{\rm{disl}}}},$$

where *η*_diff_ and *η*_disl_ are effective viscosities for diffusion and dislocation creep, respectively.

The crust is assumed to have a constant grain size, and *η*_diff_ and *η*_disl_ are computed as

6 $${\eta }_{{\rm{diff}}}=\frac{A}{2{\sigma }_{\mathrm{cr}\,}^{n-1}}\exp \left(\frac{E+PV}{RT}\right),$$

7 $${\eta }_{{\rm{disl}}}=\frac{1}{2}{A}^{\frac{1}{n}}\exp \left(\frac{E+PV}{nRT}\right){\dot{\varepsilon }}_{\,\mathrm{II(disl)}\,}^{\frac{1-n}{n}},$$

where *R* is the gas constant; *P* is pressure; *T* is temperature (in K); ${\dot{\varepsilon }}_{{\rm{II(disl)}}}=\sqrt{\frac{1}{2}{({\dot{\varepsilon }}_{ij({\rm{disl}})})}^{2}}$ is the square root of the second invariant of the strain rate tensor for dislocation creep; *σ*_cr_ is the assumed diffusion–dislocation transition stress and *A*, *E*, *V* and *n* are experimentally determined pre-exponential factor, activation energy, activation volume and stress exponent of the viscous creep, respectively, which stand for the material constant, the activation energy, the activation volume and the stress exponent, respectively.

The mantle ductile creep model takes grain size reduction and growth processes assisted by Zener pinning into account. The *η*_diff_ and *η*_disl_ are computed following refs. ^51–55^. The rheology follows a composite law in equation (5) as

8 $${\eta }_{{\rm{diff}}}=\frac{1}{2}{A}_{{\rm{diff}}}{h}^{m}\exp \left(\frac{{E}_{{\rm{diff}}}+P{V}_{{\rm{diff}}}}{RT}\right),$$

9 $${\eta }_{{\rm{disl}}}=\frac{1}{2}{A}_{\mathrm{disl}\,}^{\frac{1}{n}}\exp \left(\frac{{E}_{{\rm{disl}}}+P{V}_{{\rm{disl}}}}{nRT}\right){\dot{\varepsilon }}_{\,\mathrm{II(disl)}\,}^{\frac{1-n}{n}},$$

where *h* is the mean grain size and *m* is the grain size exponent. The interplay between diffusion and dislocation creep is controlled by a grain-size evolution equation that depends on mechanical work and temperature. The grain size evolution model is based on the following assumptions:

1. Mantle peridotite is composed of two well-mixed phases with the same density and rheology: olivine and pyroxene with a fixed volume fraction of 60% and 40%, respectively.
2. The relative motion between two phases is negligible and therefore they have the same velocity *v*.
3. The grain size distribution is close to a self-similar log-normal distribution. Therefore, it always retains the same shape, and a unique grain size (for example, the mean grain size) can fully characterize its mean variance and amplitude.

We further assume that the system is in the pinned state limit^51,55^ wherein the grain size evolution is controlled by the pinning of phases by each other (that is, Zener pinning)^51^. In these conditions, the grain size is controlled by the roughness *r* of the interface between the two phases. The relation between the mean grain size *h* and the roughness *r* is given by $h=\frac{r}{\sqrt{{h}_{{\rm{g}}}}}$, where ${h}_{{\rm{g}}}\,\approx \frac{\pi }{2}$ for the phase volume fraction in our model^55^. The roughness evolution is described by the following equations^51–53,56^:

10 $$\displaystyle\frac{dr}{dt}=\displaystyle\frac{\eta {G}_{{\rm{I}}}}{q{r}^{(q-1)}}-\displaystyle\frac{{f}_{{\rm{I}}}{r}^{2}}{\gamma _{\rm{I}} \eta }{\varPsi},$$

11 $${G}_{{\rm{I}}}=\frac{{G}_{{\rm{g}}}}{{G}_{{\rm{fac}}}}\frac{q}{p}{r}^{(q-p)},$$

12 $${G}_{{\rm{g}}}={A}_{{\rm{g}}}\exp \left(-\frac{{E}_{{\rm{g}}}+P{V}_{{\rm{g}}}}{RT}\right),$$

13 $${f}_{{\rm{I}}}={f}_{0}\exp \left(-2{\left(\frac{T}{1,000}\right)}^{2.9}\right),$$

where *G*_I_ is interface coarsening, *G*_g_ is grain growth rate, *G*_fac_ = 100 is grain growth rate factor, *q* = 4 is roughness coarsening exponent, *p* = 2 is grain size coarsening exponent, *γ*_I_ = 1 Pa m is the surface tension, *A*_g_ = 2 × 10^(4−6*p*)^ is the pre-exponential factor, *E*_g_ = 3 × 10^5^ is a grain-growth activation energy, *V*_g_ = *V*_diff_ is a grain-growth activation volume, *f*_I_ is the fraction of mechanical work *Ψ* converted to interface damage resulting in grain size reduction, *f*_0_ = 0.001 is interface damage at 1,000 K and *η* = 3*φ*_ol_*φ*_px_ is interface area density depending on the volume fractions of olivine (*φ*_ol_ = 0.6) and pyroxene (*φ*_px_ = 0.4) in the mantle.

Plastic rheology is implemented by the effective visco-plastic viscosity on the basis of the Drucker–Prager relationship with the strain-dependent strength of rocks.

14 $${\eta }_{\mathrm{visco}-\mathrm{plastic}}\le \frac{C+\mu P}{2{\dot{\varepsilon }}_{\mathrm{II}(\mathrm{visco}-\mathrm{plastic})}},$$

15 $$\mu =\left\{\begin{array}{ll}{\mu }_{0}-\gamma {\mu }_{\gamma },\quad &\,\mathrm{for}\,\gamma \le {\gamma }_{0}\\ {\mu }_{1},\quad &\,\mathrm{for}\,\gamma > {\gamma }_{0}\end{array}\right.$$

16 $$C=\left\{\begin{array}{ll}{C}_{0}-\gamma {C}_{\gamma },\quad &\,\mathrm{for}\,\gamma \le {\gamma }_{0}\\ {C}_{1},\quad &\,\mathrm{for}\,\gamma > {\gamma }_{0}\end{array}\right.$$

17 $$\gamma =\int\sqrt{\frac{1}{2}{\left({\dot{\varepsilon }}_{ij(\mathrm{plastic})}\right)}^{2}}\,{\rm{d}}t$$

where *μ* is the internal friction coefficient (*μ*_0_ and *μ*_1_ are the initial and final internal friction coefficient, respectively), *C* is the rock compressive strength at *P* = 0 (*C*_0_ and *C*_1_ are the initial and final compressive strength, respectively), *μ*_*γ*_ = (*μ*_0_ − *μ*_1_)/*γ*_0_ and *C*_*γ*_ = (*C*_0_ − *C*_1_)/*γ*_0_ are the rates of faults weakening with integrated plastic strain *γ* (*γ*_0_ is the upper strain limit for the fracture-related weakening), *t* is time (s) and ${\dot{\varepsilon }}_{ij(\mathrm{plastic})}$ is the plastic strain rate tensor.

The elastic rheology is implemented by using the Maxwell visco-elasto-plasticity model via the numerical viscosity *η*_numerical_ applied to the Stokes equation (see also Gerya^47^ for details), which depends on the numerical time step size *Δt*,

18 $${\eta }_{{\rm{numerical}}}=\frac{{\eta }_{{\rm{visco}}\mathrm{-}{\rm{plastic}}}G\Delta t}{{\eta }_{{\rm{visco}}\mathrm{-}{\rm{plastic}}}+G\Delta t},$$

where the shear modulus *G* of the crust and mantle is assumed to be 25 and 67 GPa, respectively. The corresponding visco-elasto-plastic constitutive relationship is written as

19 $${\boldsymbol{\tau }}=2{\eta }_{{\rm{numerical}}}{\dot{\boldsymbol{\varepsilon}}}_{{\rm{total}}}+\frac{{\eta }_{{\rm{numerical}}}}{G\Delta t}{{\boldsymbol{\tau }}}_{0},$$

where ${\dot{\boldsymbol{\varepsilon}}}_{{\rm{total}}}$ is the total deviatoric strain rate and **τ**_0_ indicates the deviatoric stress of the previous numerical time step.

### Topographic features

To compare the modelled and observed topography, we extract a set of profiles from both datasets using a sampling size of 16 km, consistent with the global resolution of Magellan observations. For the modelled results, the surface topography is downsampled by block averaging over the non-overlapping squared windows. For the observations, the topography is derived from the VenusTopo719 model^26^ within the regions shown in Fig. 5, covering the same lateral extent as the model domain. For each rift, a total of 32 profiles are extracted perpendicular to the extension of the rift valley to characterize their topographic features. Each profile is independently processed, and the medium, 25th and 75th percentiles of the following topographic features throughout the rift are used to characterize their typical values (Extended Data Table 3).

Before extracting topographic features, the quadratic background trend is removed, which is fitted using the outermost 256 km at both ends of the profile. The valley bottom is defined as the minimum across the profile. On either side with respect to the bottom, the peak of the uplift flank is defined as the highest elevation with respect to the bottom. The horizontal distance between the two flank peaks is the valley width. The valley elevation offset is defined as the difference in elevation between the highest peak and the lowest point. Moving outwards from the peak of each flank, the outer boundary of the uplifted flank is defined as the first location where the elevation decreases below the root mean square elevation on the subsequent 96-km segment. This corresponds to the location where the flank topography decays into the background roughness level and is thus indistinguishable from the surrounding terrain. The width of the flank uplift is defined as the horizontal distance between the peak and the boundary (examples are shown in Extended Data Fig. 7d–f). The results shown are statistics for the average flank width of each profile. We note that increasing or decreasing the baseline for deriving the background topographic roughness by a factor of two varies the measured widths of flank uplifts from observations by only one or two sampling sizes, which does not affect the conclusions. The topographic roughness is equal to or greater than the model resolution; therefore, the identification of the rift flank uplift boundary is inherently limited by the model resolution.

## Online content

Any methods, additional references, Nature Portfolio reporting summaries, source data, extended data, supplementary information, acknowledgements, peer review information; details of author contributions and competing interests; and statements of data and code availability are available at https://doi.org/10.1038/s41561-026-02044-8.

## Data Availability

The topographic model VenusTopo719 (ref. ^26^) is available via Zenodo at https://zenodo.org/records/3870926 (ref. ^45^). The perceptually uniform colour maps^57^ used in this paper are available via Zenodo at https://zenodo.org/records/8409685 (ref. ^58^). The data for the viscosity structure, surface topography and Moho relief presented in this study are available via Zenodo at https://doi.org/10.5281/zenodo.20156810 (ref. ^59^).

## Extended data

**Extended Data Table 1:** Summary of the conditions and results of the numerical experiments

Extension rate	Rheology of crust		
	Plagioclase	Dry diabase	Mafic granulite
150-km-thick lithosphere (~1650 K isotherm) and 20-km-thick crust			
1 cm/yr	P1 Regime 1	D1 Regime 2	G1 Regime 2
3 cm/yr	P2 Regime 2	D2 Regime 2	G2 Regime 3
10 cm/yr	P3 Regime 2	D3 Regime 3	G3 Regime 3
100-km-thick lithosphere (~1625 K isotherm) and 30-km-thick crust			
10 cm/yr	P4 Regime 1	D4 Regime 1	G4 Regime 1

Regime 1: The models that do not form a rift valley with a clear fault-like feature. Regime 2: The models that form a rift valley with wide flank uplifts but without a dominant normal or detachment fault. Regime 3: The models that form a rift valley with wide flank uplifts and at least a dominant normal or detachment fault.

**Extended Data Table 2:** Physical properties of rocks used in numerical experiments

Material	*ρ*_0_ (kg/m ^3^)	Thermal conductivity (W/m/K at *T*)	Flow law
Plagioclase, An75 ^a^	2800	$1.18+\frac{474}{T+77}$	$A=4.80\times {10}^{22}\,{\rm{P}}{{\rm{a}}}^{n}{\rm{s}},n=3.2$, $E=238000\,{\rm{J}}/{\rm{mol}},V=0\,{\rm{J}}/{\rm{mol}}/{\rm{MPa}}$, ${{{\sigma}}}_{\mathrm{cr}}=3\times {10}^{4}\,{\rm{Pa}}$, ${C}_{0}=10\,{\rm{MPa}},{C}_{1}=1\,{\rm{MPa}}$, ${{\rm{\mu }}}_{0}=0.6,{{\rm{\mu }}}_{1}=0.15$
Dry diabase ^a^	2800	$1.18+\frac{474}{T+77}$	$A=1.26\times {10}^{24}\,{\rm{P}}{{\rm{a}}}^{n}{\rm{s}},n=3.2$, $E=260000\,{\rm{J}}/{\rm{mol}},V=0\,{\rm{J}}/{\rm{mol}}/{\rm{MPa}}$, ${\sigma }_{\mathrm{cr}}=3\times {10}^{4}\,{\rm{Pa}}$, ${C}_{0}=10\,{\rm{MPa}},{C}_{1}=1\,{\rm{MPa}}$, ${\mu }_{0}=0.6,{\mu }_{1}=0.15$
Mafic granulite ^a^	2800	$1.18+\frac{474}{T+77}$	$A=1.13\times {10}^{21}\,{\rm{P}}{{\rm{a}}}^{n}{\rm{s}},n=3.2$, $E=445000\,{\rm{J}}/{\rm{mol}},V=0\,{\rm{J}}/{\rm{mol}}/{\rm{MPa}}$, ${\sigma }_{\mathrm{cr}}=3\times {10}^{4}\,{\rm{Pa}}$, ${C}_{0}=10\,{\rm{MPa}},{C}_{1}=1\,{\rm{MPa}}$, ${\mu }_{0}=0.6,{\mu }_{1}=0.15$
Dry olivine ^b^	3300	$0.73+\frac{1293}{T+77}$	$m=3,{A}_{\mathrm{diff}}=6.67\times {10}^{14}\,{\rm{Pa\; s}}$, ${E}_{\mathrm{diff}}=375000\,{\rm{J}}/{\rm{mol}}$, ${V}_{\mathrm{diff}}=10\,{\rm{J}}/{\rm{mol}}/{\rm{MPa}}$, ${A}_{\mathrm{disl}}=9.09\times {10}^{15}\,{\rm{P}}{{\rm{a}}}^{n}{\rm{s}},n=3.5$, ${E}_{\mathrm{disl}}=530000\,{\rm{J}}/{\rm{mol}}$, ${V}_{\mathrm{disl}}=14\,{\rm{J}}/{\rm{mol}}/{\rm{MPa}}$, ${C}_{0}=10\,{\rm{MPa}},{C}_{1}=1\,{\rm{MPa}}$, ${{\rm{\mu }}}_{0}=0.6,{{\rm{\mu }}}_{1}=0.15$

The thermal conductivity is a function of temperature in K. The flow law parameters describe the viscous and plastic properties (see Methods).

^a^ Ranali, 1995 (ref. ^22^).

^b^ Hirtha and Kohlstedt, 2003 (ref. ^23^).

**Extended Data Table 3:** Summary of rift topographic features derived with the 16 km sampling size

Model	Time (Myr)	Valley width (km)	Flank width (km)	Elevation offset (km)	Figure
P1	8.9	144 (144–160)	64 (64–72)	2.3 (1.9–2.4)	ED Fig. 3d
P2	3.0	168 (128–180)	72 (64–80)	3.7 (3.3–3.8)	ED Fig. 3e
P3	0.7	160 (128–176)	96 (88–112)	2.4 (2.1–2.8)	Fig. 2f
	0.9	176 (156–192)	96 (88–104)	4.3 (3.6–5.1)	ED Fig. 3f
D1	9.0	128 (124–132)	72 (64–72)	2.8 (2.7–3.0)	Fig. 3d
D2	3.0	144 (112–176)	72 (64–88)	5.0 (4.0–5.4)	Fig. 3e
D3	0.7	160 (144–176)	120 (112–178)	4.0 (3.7–4.6)	Fig. 2e
	0.9	160 (128–176)	104 (104–128)	6.0 (5.7–6.4)	Fig. 3f
D3 ^a^	1.8	160 (120–160)	80 (56–88)	3.5 (3.2–3.9)	ED Fig. 5d
	15.6	144 (96–160)	32 (24–40)	2.5 (2.2–2.9)	ED Fig. 5e
	105.6	144 (120–160)	24 (16–24)	1.7 (1.5–1.9)	ED Fig. 5f
G1	8.9	128 (112–128)	64 (56–72)	4.8 (4.6–5.0)	ED Fig. 2d
G2	1.4	128 (128–144)	88 (64–104)	1.3 (1.2–1.3)	Fig. 1d
	2.1	96 (96–100)	128 (120–128)	6.3 (5.6–6.6)	Fig. 1e
	2.7	128 (128–128)	104 (104–112)	7.2 (6.6–7.9)	Fig. 1f
	3.1	128 (128–128)	88 (88–96)	6.2 (5.9–6.6)	ED Fig. 2e
G2 ^b^	5.0	96 (96–100)	80 (80–88)	5.4 (4.6–5.7)	ED Fig. 6d
	28.2	96 (96–96)	56 (48–64)	4.6 (4.1–4.8)	ED Fig. 6e
	103.1	96 (96–96)	48 (40–48)	4.2 (3.8–4.3)	ED Fig. 6f
G3	0.7	128 (128–132)	168 (160–184)	8.0 (7.5–9.1)	Fig. 2d
	0.9	144 (144–160)	136 (128–168)	7.5 (6.5–8.2)	ED Fig. 2f
Dali		192 (192–224)	112 (104–122)	4.7 (4.1–5.2)	ED Fig. 7a
Ganis		192 (160–212)	160 (88–192)	4.0 (3.4–4.6)	ED Fig. 7b
Devana		192 (176–224)	184 (176–200)	3.8 (3.3–4.1)	ED Fig. 7c

Statistic values are reported as median with 25th and 75th percentiles shown in parenthesis. Extended data figures are denoted by ED.

^a^ Relaxation starts at 0.7 Myr

^b^ Relaxation starts at 2.1 Myr.

## Code availability

The visco-elasto-plastic version of the I3ELVIS code used in this study is available from the corresponding author on request.

## References

[1] O’Rourke, J. G. et al. Venus, the planet: introduction to the evolution of Earth’s sister planet. *Space Sci. Rev.* **219**, 10 (2023).

[2] Price, M. & Suppe, J. Constraints on the resurfacing history of Venus from the hypsometry and distribution of volcanism, tectonism, and impact craters. *Earth Moon Planets* **71**, 99–145 (1995).

[3] Ivanov, M. A. & Head, J. W. Global geological map of Venus. *Planet. Space Sci.* **59**, 1559–1600 (2011).

[4] Stofan, E. R., Smrekar, S. E., Bindschadler, D. L. & Senske, D. A. Large topographic rises on Venus: implications for mantle upwelling. *J. Geophys. Res.* **100**, 23317 (1995).

[5] Guseva, E. N. Classification of the rift zones of venus: rift valleys and graben belts. *Sol. Syst. Res.* **50**, 184–196 (2016).

[6] Smrekar, S. E. et al. Recent hotspot volcanism on Venus from VIRTIS emissivity data. *Science* **328**, 605–608 (2010). 20378775 10.1126/science.1186785

[7] Piskorz, D., Elkins Tanton, L. T. & Smrekar, S. E. Coronae formation on Venus via extension and lithospheric instability. *J. Geophys. Res. Planets* **119**, 2568–2582 (2014).

[8] Gülcher, A. J. P., Sabbeth, L., Stofan, E. R. & Smrekar, S. E. Coronae on Venus: an updated global database and insights into morphology, spatial distribution, geological setting, and lithospheric properties. *J. Geophys. Res. Planets* **130**, 2024–008749 (2025).

[9] Ghail, R. C. et al. Volcanic and tectonic constraints on the evolution of Venus. *Space Sci. Rev.* **220**, 36 (2024).

[10] McGill, G. E., Steenstrup, S. J., Barton, C. & Ford, P. G. Continental rifting and the origin of Beta Regio, Venus. *Geophys. Res. Lett.* **8**, 737–740 (1981).

[11] Foster, A. & Nimmo, F. Comparisons between the rift systems of East Africa, Earth and Beta Regio, Venus. *Earth Planet. Sci. Lett.* **143**, 183–195 (1996).

[12] Stoddard, P. R. & Jurdy, D. M. Topographic comparisons of uplift features on Venus and Earth: implications for Venus tectonics. *Icarus* **217**, 524–533 (2012).

[13] Graff, J. R., Ernst, R. E. & Samson, C. Evidence for triple-junction rifting focussed on local magmatic centres along Parga Chasma, Venus. *Icarus* **306**, 122–138 (2018).

[14] Zuber, M. T. & Parmentier, E. M. Lithospheric necking: a dynamic model for rift morphology. *Earth Planet. Sci. Lett.* **77**, 373–383 (1986).

[15] Liao, J. & Gerya, T. From continental rifting to seafloor spreading: insight from 3D thermo-mechanical modeling. *Gondwana Res.* **28**, 1329–1343 (2015).

[16] Koptev, A., Calais, E., Burov, E., Leroy, S. & Gerya, T. Dual continental rift systems generated by plume–lithosphere interaction. *Nat. Geosci.* **8**, 388–392 (2015).

[17] Sternai, P. Surface processes forcing on extensional rock melting. *Sci. Rep.* **10**, 7711 (2020). 32382159 10.1038/s41598-020-63920-w PMC7206043

[18] Banerdt, W. B. & Golombek, M. P. Deformational models of rifting and folding on Venus. *J. Geophys. Res. Solid Earth* **93**, 4759–4772 (1988).

[19] Solomon, S. C. & Head, J. W. Rift structures on Venus: implications of a lithospheric stretching model. In *Lunar and Planetary Science Conference* 806–807 (1984).

[20] Regorda, A. et al. Rifting Venus: insights from numerical modeling. *J. Geophys. Res. Planets* **128**, 2022–007588 (2023).

[21] Gülcher, A. J. P., Gurnis, M. & Smrekar, S. E. Dynamics of Venusian rifts and their interactions with plumes and intrusions. *Earth Planet. Sci. Lett.* **667**, 119514 (2025).

[22] Ranalli, G. *Rheology of the Earth* (Springer, 1995).

[23] Hirth, G. & Kohlstedt, D. in *Geophysical Monograph Series* Vol. 138 (ed Eiler, J.) 83–105 (American Geophysical Union, 2003); 10.1029/138GM06

[24] Smrekar, S. E., Ostberg, C. & O’Rourke, J. G. Earth-like lithospheric thickness and heat flow on Venus consistent with active rifting. *Nat. Geosci.* **16**, 13–18 (2023).

[25] Maia, J. S. et al. The seismogenic thickness of venus. *J. Geophys. Res. Planets* **130**, 2025–009065 (2025).

[26] Wieczorek, M. A. Gravity and topography of the terrestrial planets. *Treatise Geophys.* **10**, 165–206 (2015).

[27] Sandwell, D. T., Johnson, C. L., Bilotti, F. & Suppe, J. Driving forces for limited tectonics on Venus. *Icarus* **129**, 232–244 (1997).

[28] Guseva, E. N. & Ivanov, M. A. Regional geologic and morphologic characterization of rift zones on Venus. *Sol. Syst. Res.* **53**, 233–244 (2019).

[29] Shalygin, E. V. et al. Active volcanism on Venus in the Ganiki Chasma rift zone. *Geophys. Res. Lett.* **42**, 4762–4769 (2015).

[30] Brossier, J., Gilmore, M. S. & Head, J. W. Extended rift-associated volcanism in Ganis Chasma, Venus detected from Magellan radar emissivity. *Geophys. Res. Lett.* **49**, 2022–099765 (2022).

[31] Mackwell, S. J., Zimmerman, M. E. & Kohlstedt, D. L. High-temperature deformation of dry diabase with application to tectonics on Venus. *J. Geophys. Res. Solid Earth* **103**, 975–984 (1998).

[32] Nimmo, F. & Mackwell, S. Viscous relaxation as a probe of heat flux and crustal plateau composition on Venus. *Proc. Natl Acad. Sci. USA* **120**, 2216311120 (2023). 10.1073/pnas.2216311120 PMC9934203 36623181

[33] Brune, S., Williams, S. E., Butterworth, N. P. & Müller, R. D. Abrupt plate accelerations shape rifted continental margins. *Nature* **536**, 201–204 (2016). 27437571 10.1038/nature18319

[34] Lourenço, D. L., Rozel, A. B., Ballmer, M. D. & Tackley, P. J. Plutonic squishy lid: a new global tectonic regime generated by intrusive magmatism on Earth like planets. *Geochem. Geophys. Geosyst.* **21**, 2019–008756 (2020).

[35] Byrne, P. K. et al. A globally fragmented and mobile lithosphere on Venus. *Proc. Natl Acad. Sci. USA* **118**, 2025919118 (2021). 10.1073/pnas.2025919118 PMC8255999 34155105

[36] Gerya, T. V., Perchuk, L. L., Maresch, W. V. & Willner, A. P. in *Gneiss Domes in Orogeny* (eds Whitney, D. L., Teyssier, C. & Siddoway, C. S.) 97–115 (Geological Society of America, 2004); 10.1130/0-8137-2380-9.97

[37] Maia, J. S. & Wieczorek, M. A. Lithospheric structure of Venusian crustal plateaus. *J. Geophys. Res. Planets* **127**, 2021–007004 (2022).

[38] Anderson, F. S. & Smrekar, S. E. Global mapping of crustal and lithospheric thickness on Venus. *J. Geophys. Res. Planets* **111**, 2004–002395 (2006).

[39] Gülcher, A. J. P., Gerya, T. V., Montési, L. G. J. & Munch, J. Corona structures driven by plume–lithosphere interactions and evidence for ongoing plume activity on Venus. *Nat. Geosci.* **13**, 547–554 (2020).

[40] Cascioli, G., Gülcher, A. J. P., Mazarico, E. & Smrekar, S. E. A spectrum of tectonic processes at coronae on Venus revealed by gravity and topography. *Sci. Adv.* **11**, 5932 (2025). 10.1126/sciadv.adt5932 PMC12077499 40367154

[41] Simons, M., Solomon, S. C. & Hager, B. H. Localization of gravity and topography: constraints on the tectonics and mantle dynamics of Venus. *Geophys. J. Int.* **131**, 24–44 (1997).

[42] Gerya, T. & Burov, E. Nucleation and evolution of ridge–ridge–ridge triple junctions: thermomechanical model and geometrical theory. *Tectonophysics* **746**, 83–105 (2018).

[43] Koptev, A., Gerya, T., Calais, E., Leroy, S. & Burov, E. Afar triple junction triggered by plume-assisted bidirectional continental break-up. *Sci. Rep.* **8**, 14742 (2018). 30283091 10.1038/s41598-018-33117-3 PMC6170478

[44] Carter, L. M. et al. Sedimentary processes on Venus. *Space Sci. Rev.* **219**, 85 (2023).

[45] Wieczorek, M. A. Spherical harmonic model of the planet Venus: VenusTopo719. *Zenodo*10.5281/zenodo.3870926 (2015).

[46] Gerya, T. V. & Yuen, D. A. Robust characteristics method for modelling multiphase visco-elasto-plastic thermo-mechanical problems. *Phys. Earth Planet. Inter.* **163**, 83–105 (2007).

[47] Gerya, T. *Introduction to Numerical Geodynamic Modelling* (Cambridge Univ. Press, 2019).

[48] Gerya, T. New i3elvis: robust visco-elasto-plastic geodynamic modelling code based on staggered finite differences and marker in cell. In *EGU General Assembly Conference Abstracts* 22–13215 (2022); 10.5194/egusphere-egu22-13215

[49] Gerya, T. V. Three-dimensional thermomechanical modeling of oceanic spreading initiation and evolution. *Phys. Earth Planet. Inter.* **214**, 35–52 (2013).

[50] Duretz, T., De Borst, R. & Le Pourhiet, L. Finite thickness of shear bands in frictional viscoplasticity and implications for lithosphere dynamics. *Geochem. Geophys. Geosyst.* **20**, 5598–5616 (2019).

[51] Bercovici, D. & Ricard, Y. Mechanisms for the generation of plate tectonics by two-phase grain-damage and pinning. *Phys. Earth Planet. Inter.* **202–203**, 27–55 (2012).

[52] Mulyukova, E. & Bercovici, D. Formation of lithospheric shear zones: effect of temperature on two-phase grain damage. *Phys. Earth Planet. Inter.* **270**, 195–212 (2017).

[53] Bercovici, D. & Ricard, Y. Generation of plate tectonics with two-phase grain-damage and pinning: source–sink model and toroidal flow. *Earth Planet. Sci. Lett.* **365**, 275–288 (2013).

[54] Bercovici, D. & Ricard, Y. Plate tectonics, damage and inheritance. *Nature* **508**, 513–516 (2014). 24717430 10.1038/nature13072

[55] Bercovici, D., Schubert, G. & Ricard, Y. Abrupt tectonics and rapid slab detachment with grain damage. *Proc. Natl Acad. Sci. USA* **112**, 1287–1291 (2015). 25605890 10.1073/pnas.1415473112 PMC4321316

[56] Rozel, A., Ricard, Y. & Bercovici, D. A thermodynamically self-consistent damage equation for grain size evolution during dynamic recrystallization. *Geophys. J. Int.* **184**, 719–728 (2011).

[57] Crameri, F., Shephard, G. E. & Heron, P. J. The misuse of colour in science communication. *Nat. Commun.* **11**, 5444 (2020). 33116149 10.1038/s41467-020-19160-7 PMC7595127

[58] Crameri, F. Scientific colour maps. *Zenodo*10.5281/zenodo.8409685 (2023).

[59] Yang, X., Gerya, T. & Gülcher, A. Thermomechanical modeling data for Venusian rifting. *Zenodo*10.5281/zenodo.20156810 (2026).

